# Design, Synthesis and Bioactivity of *N*-Glycosyl-*N′*-(5-substituted phenyl-2-furoyl) Hydrazide Derivatives

**DOI:** 10.3390/ijms15046741

**Published:** 2014-04-21

**Authors:** Zining Cui, Hang Su, Jiazhen Jiang, Xinling Yang, Yoshihiro Nishida

**Affiliations:** 1 Guangdong Province Key Laboratory of Microbial Signals and Disease Control, Department of Plant Pathology, College of Natural Resources and Environment, South China Agricultural University, Guangzhou 510642, China; 2 Division of Nanobiology, Advanced Integrated Science, Chiba University, Matsudo, Chiba 271-0092, Japan; E-Mail: ynishida@faculty.chiba-u.jp; 3 Department of Applied Chemistry, College of Science, China Agricultural University, Beijing 100193, China; E-Mails: kongkong_1989@sina.cn (H.S.); yangxl@cau.edu.cn (X.Y.); 4 State Key Laboratory for Biology of Plant Diseases and Insect Pests, Institute of Plant Protection, Chinese Academy of Agricultural Sciences, Beijing 100193, China

**Keywords:** glycosylhydrazide, synthesis, tautomer, antifungal activity, antitumor activity

## Abstract

Condensation products of 5-substituted phenyl-2-furoyl hydrazide with different monosaccharides d-glucose, d-galactose,d-mannose, d-fucose and d-arabinose were prepared. The anomerization and cyclic-acyclic isomers were investigated by ^1^H NMR spectroscopy. The results showed that, except for the d-glucose derivatives, which were in the presence of β-anomeric forms, all derivatives were in an acyclic Schiff base form. Their antifungal and antitumor activities were studied. The bioassay results indicated that some title compounds showed superior effects over the commercial positive controls.

## 1. Introduction

Carbohydrates participate in various vital processes, showing important physiological and biological activities. In a recent review, Becker and coworkers [1] emphasized that the introduction of pharmacophores into sugar templates that possess dense stereochemical information is an excellent strategy for the development of bioactive compounds with rich structural diversity. Free reducing sugars (hemiacetal), which are often available from natural sources, react with α-heteroatom nucleophile hydrazide to give cyclic pyranoside adducts in predominantly β-anomeric form or the open chain hydrazone tautomer [2–5]. In fact, this intrinsic chemoselective condensation reactivity of carbohydrates has been skillfully employed in numerous applications, including biotin labeling [6–8], formation of glycoarrays [9–11], glycan capture for structural and functional glycomics [12–14], and the generation of glycopeptide analogues [15,16].

Hydrazones containing an azometine (Schiff base) –NHN=CH– group possess various bioactivities such as antimicrobial, anticonvulsant, analgesic, antiinflammatory, antiplatelet, antitubercular and antitumoral activities [17]. A very famous example is the isonicotinic acid hydrazide (isoniazid) which showed very high inhibitory activity towards *Mycobacterium tuberculosis* H37Rv *in vivo*. Researchers synthesized isoniazid-hydrazones derivatives that were reported to have inhibitory activity in mice infected with various strains of *M. tuberculosis* and also showed less toxicity in these mice than isoniazid [18] because of the blockage of –NH_2_ group. These findings further support the growing importance of the synthesis of hydrazide-hydrazones compounds [19].

**Scheme 1.**
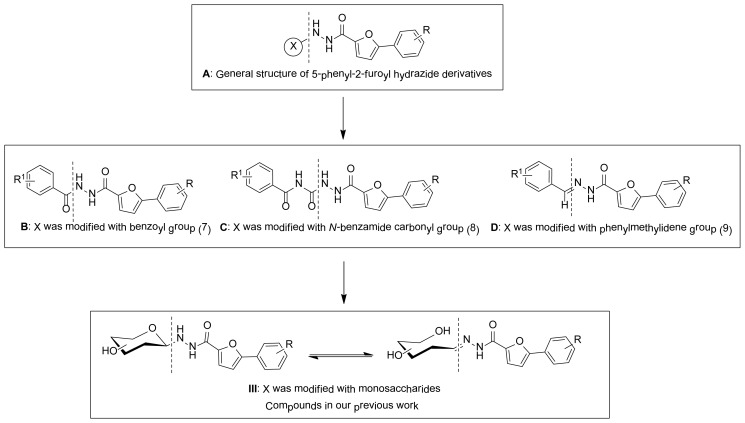
Designed strategy for title compounds.

In our previous work, based on the hydrazide moiety, some diacylhydrazines (**B**, Scheme 1) [20–24], semicarbazides (**C**, Scheme 1) [25], and acylhydrazones (**D**, Scheme 1) [26,27] derivatives containing 5-phenyl-2-furan were designed and synthesized. All the compounds showed diverse and significant bioactivities such as fungicidal, insecticidal, and antitumor activities. In continuation of our research on the synthesis of biological heterocyclic compounds, a series of novel glycosyl hydrazide derivatives containing 5-phenyl-2-furan moiety were designed and synthesized (Scheme 1). The ring-chain isomers were investigated by ^1^H NMR spectroscopy. Their antifungal and antitumor activities were evaluated.

## 2. Results and Discussion

### 2.1. Synthesis and Structure Elucidation

**Scheme 2.**
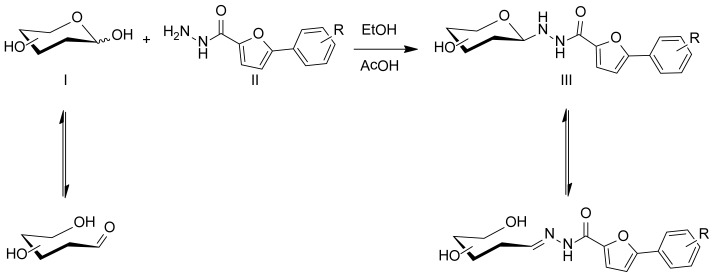
Condensation reactivity and equilibrium between cyclic and acyclic forms of the monosaccharide. **III-1:** Glc, R = 4-Cl; **III-2:** Gal, R = 4-Cl; **III-3:** Man, R = 4-Cl; **III-4:** Fuc, R = 4-Cl; **III-5:** Ara, R = 4-Cl; **III-6:** Glc, R = 2,4-di-F; **III-7:** Glc, R = 4-F; **III-8:** Glc, R = 4-NO_2_; **III-9:** Glc, R = 2-NO_2_; **III-10:** Glc, R = 2-Cl; **III-11:** Glc, R = 4-Me; **III-12:** Glc, R = 3-F.

Glycosylhydrazides were obtained by condensation of equimolar amounts of the corresponding 5-substituted phenyl-2-furoyl hydrazide with the monosaccharides d-glucose, d-galactose, d-mannose, d-fucose, and d-arabinose in ethanol (Scheme 2).

The structures of all the compounds were characterized by ^1^H Nuclear Magnetic Resonance (NMR), Infrared Spectroscopy (IR) and High Resolution Mass Spectrometer (HRMS). In the IR spectra, the compounds showed absorption bands around 3200 to 3400 cm^−1^ originating from the O–H and N–H stretching vibration. The strong bands around 1640 to 1680 cm^−1^ were carbonyl vibration of the secondary amide. The bands between 1600 and 1620 cm^−1^ could be assigned to the C=N stretching vibration. Absorption bands around 1610, 1550 and 1475 cm^−1^ were attributed to the frame vibration of the phenyl and furan ring. Absorption bands around 1430, 1330 and 1260 cm^−1^ were attributed to the coupled modes of C–C and C–O stretching vibrations of the sugar residues [28,29]. The absorption bands around the 1150 and 1085 cm^−1^ region are characteristic for the pyranose form of hydrazines, and those around the 1075 cm^−1^ region for the acyclic form of hydrazones [2].

In the ^1^H NMR spectra, the ring-chain tautomers of the monosaccharides were obviously determined by the chemical shift of the proton in secondary amide CO–NH and the proton in imine N=CH in DMSO-*d*_6_ solution (Table 1).

**Figure 1.**
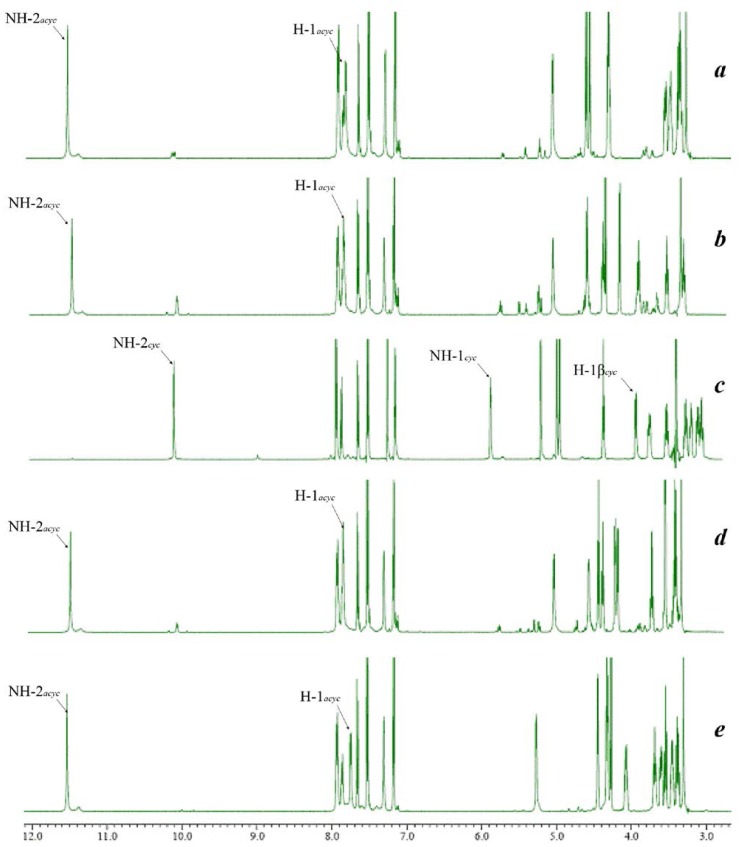
500 MHz ^1^H NMR spectra of condensation products of 5-(4′-chlorophenyl)-2-furoyl hydrazide with different monosaccharides: d-arabinose (**a**); d-fucose (**b**); d-glucose (**c**); d-galctose (**d**); and d-mannose (**e**) in DMSO-*d*_6_ solution.

If the isomer was a cyclic form, the signal of the amide CO–NH was appeared around 10.20 ppm and there was no imine N=CH signal, meanwhile the other N–NH signal was appeared around 5.87 ppm (Figure 1c). If the isomer was an acyclic form, the signal of the amide CO–NH was down-fileded to 11.50 ppm and the imine N=CH signal was appeared around 7.75 ppm (Figure 1e). The result showed that except the d-Glc, other monosaccharides such as d-Gal, d-Man, d-Fuc and d-Ara hydrazide derivatives were mainly the acyclic tautomers (Figure 1). The chemical shift of the anomeric H-1 in glucose 3.89 ppm and the coupling constant of 8.25 Hz indicated that the glucose was in the presence of β isomer.

The ring-chain interconversion of sugar hydrazones in solution usually depended on factors such as the sugar conformation, most probably on the proportion of aldehyde form in solution, the basicity of the hydrazine groups, and the solvent effect of proton-acceptor ability. About the solvent effect on ring-chain interconversion, there were evidence that sugar hydrazones were favorable to be acyclic form in dimethyl sulfoxide-*d*_6_ solution than others, such as pyridine-*d*_5_, methanol-*d*_4_ and deuterium oxide [2]. High pH values also stimulated the ring to chain conversion. The cyclic to acyclic conversion caused by increase in the basicity of hydrazine and the proton-acceptor ability of solvent might be interpreted according to the electronic theory. The strongly basic nitrogen atom of hydrazine group was in high electron-density. The electron could move easily from nitrogen to anomeric carbon of the sugars to form a C=N bong with *sp*^2^ hybridized orbital. The solvent with strong proton-acceptor ability could increase the basicity of the hydrazine group owing to the proton bonding with N–H group. In our study, the same solution and hydrazine were used (Figure 1), suggesting that the ring-chain tautomer was mainly caused due to the different monosaccharides. Some research [2] showed that glucose was prone to be cyclic form especially in dimethylsulfoxide (DMSO) solution. In this study, only glucose derivatives gave the cyclic form, which was consistent with the reported result [2]. Although the tautomerism was analyzed by ^1^H NMR, these were only the states in DMSO solution and did not reflect in any other solutions or in physiological environment. The equilibrium could move in other solutions, especially under the physiological situation.

### 2.2. Bioassay

#### 2.2.1. Fungicidal Activity

The fungicidal activity and fungicidal spectra were evaluated by preliminary bioassay against 19 kinds of fungi (Table 2).

The result showed that the title compounds had broad fungicidal spectra, and most of them possessed good activity against the tested fungi at 50 μg·mL^−1^. Compound **III-5** (Arabinose derivative) showed good activity against *Phytophthora infestans* and moderate activity against *Phomopsis aspamgi* and *Cladosporium fulvum*. Compound **III-4** (Fucose derivative) showed moderate activity against *P. aspamgi*. Compound **III-3** (Mannose derivative) showed good activity against *P. infestans* and *Alternaria alternata*. All the compounds had no activity against the *Sclerotinia sclerotiorum* except compound **III-2** (Galactose derivative), which also showed excellent activity against the *P. infestans*, and moderate activity against *A. alternate* and *P. aspamgi*. For the glucose derivatives, fluorinated compounds such as **III-6** (2,4-di-F), **III-7** (4-F), and **III-12** (3-F) showed better activity than the others. Among them, compound **III-6** exhibited excellent activity against *P. infestans* and compound **III-7** showed excellent activity against *P. aspamgi*. Both compounds **III-6** and **III-12** showed a significant inhibition effect on *Valsa mali* and *C. fulvum*. Compound **III-12** also showed good activity against *A. alternate* and *P. aspamgi*. Compounds **III-1** and **III-9** showed considerable activity against *Phytophthora melonis*. Compound **III-11** showed some inhibitory effect on the *P. melonis*. All the compounds showed poor activities against *Fusarium graminearum*, *Pyricularia oryzae*, *Monilinia ariae*, *Gloeosporium musarum*, *Fusarium oxysporum* f. sp. Niveum, *Botrytis cinerea* Pers., *Colletotrichum orbiculare*, *Alternaria dauci* and *Colletotrichum phomoides*.

EC_50_ and EC_80_ values against some fungi were studied and showed in Table 3. The EC_50_ values of **III-2**, **III-5**, **III-8** against *P. infestans* were 4.493, 5.476, and 5.695 μg·mL^−1^, respectively, which were better than that of the positive control carbendazim (EC_50_ = 5.943 μg·mL^−1^). Among them, especially the compound **III-8**, which EC_80_ value of 195.839 μg·mL^−1^ was also better than that of the positive carbendazim (EC_80_ = 219.690 μg·mL^−1^). The EC_50_ and EC_80_ values of **III-11** against *Alternaria tenuis* Nees were 6.181 and 431.342 μg·mL^−1^, respectively, which were better than that of the positive control thiram (EC_50_ = 8.831 μg·mL^−1^ and EC_80_ = 608.260 μg·mL^−1^). The EC_50_ of **III-7** against *Colletotrichum gloeosporioides* was 4.962 μg·mL^−1^, which was close to that of the positive control carbendazim (EC_50_ = 4.613 μg·mL^−1^), while its EC_80_ value of 210.254 μg·mL^−1^ was better than that of the positive control (EC_80_ = 352.820 μg·mL^−1^). Also for this compound, its EC_50_ against *P. aspamgi* was 2.737 μg·mL^−1^, which was better than that of hymexazol (EC_50_ = 3.656 μg·mL^−1^), while their EC_80_ was the same with each other. The EC_50_ values of **III-1** and **III-9** against *P. melonis* were 5.179 and 7.586 μg·mL^−1^. The activity of compound **III-1** was close to that of the positive control mancozeb (EC_50_ = 5.408 μg·mL^−1^) and better than compound **III-9**. Although their EC_80_ values of 840.493 and 500.113 μg·mL^−1^ were far away from that of the positive control mancozeb (EC_80_ = 211.870 μg·mL^−1^), meanwhile the EC_80_ value of compound **III-9** was much better than compound **III-1**.

#### 2.2.2. Antitumor Activity

Antitumor activity of title compounds was checked. The antitumor activity in Table 4 showed that some title compounds had great activity against human promyelocytic leukemic cells (HL-60). In which, the activity of compounds **III-3**, **III-4** and **III-5** (IC_50_ = 6.9, 1.2, and 19.4 μM) was better than that of the positive control doxorubicin (IC_50_ = 28.4 μM). Some compounds exhibited good activity against human gastric carcinoma cells (BGC-823), such as **III-4**, **III-5**, and **III-6** (IC_50_ = 6.9, 1.2, and 19.4 μM), but which were lower than that of the doxorubicin (IC_50_ = 8.5 μM). Against human nasopharyngeal carcinoma cells (KB), only compound **III-3** showed better activity (IC_50_ = 9.0 μM) than the positive control doxorubicin (IC_50_ = 28.4 μM). All the compounds showed poor activity against human hepatocellular carcinoma cells (Bel-7402).

The preliminary structure-activity relationship analysis indicated that compounds containing Man (**III-3**), Fuc (**III-4**), and Ara (**III-5**) showed better activity against human promyelocytic leukemic cells (HL-60). Although the compounds containing glucose showed poorer activity against HL-60 than doxorubicin, some of them also possessed considerable activity, and the substituted group had obvious effects on the activity. The compounds with fluoride and nitro group on the *ortho* (**III-6** and **III-9**) or *para-*position (**III-7** and **III-8**) showed better activity than the others. It could be deduced that the skeleton of 5-phenyl-2-furoyl hydrazide (hydrazone) displayed important antitumor activity, which was in agreement with our former results [20–24,26,27].

## 3. Experimental Section

### 3.1. General Information

All the melting points were determined with a Cole-Parmer melting point apparatus while the thermometer was uncorrected. IR spectra were recorded on a NEXUS-470 FTIR (Nicolet) spectrometer with KBr pellets. ^1^H NMR spectra were recorded with Bruker DPX300 (Bruker, Billerica, MA, USA) and JEOL JNM-ECA500 instrument (JEOL Ltd., Tokyo, Japan), while tetramethylsilane was used as the internal standards. HRMS was performed with Bruker APEX IV instrument (Bruker, Billerica, MA, USA). Analytical thin-layer chromatography (TLC) was carried out on precoated plates (silica gel 60 F254). The developing solvents were chloroform and methanol (*v/v*: 6/1), and spots were visualized with ultraviolet (UV) light.

### 3.2. Synthetic Procedures

#### 3.2.1. General Synthetic Procedure for the Key Intermediates

Intermediates **II** were synthesized from substituted aniline by Meerwein arylation reaction using the reported procedure [20–27].

#### 3.2.2. General Synthetic Procedure for the Title Compounds **III**

A mixture of monosaccaride **I** (0.05 mol) and 5-substituted phenyl-2-furoyl hydrazide **II** (0.05 mol) reacted in ethanol under reflux for 6–8 h. The reaction was catalyzed by drops of acetic acid. After cooling, the solvent was removed under reduced pressure, and the solid was recrystallized from ethanol to obtain the target compounds **III**.

All the synthesized compounds were solid. Their structures were confirmed by ^1^H NMR, IR, and ESI-HRMS.

***N*-(β-d-Glucopyranosyl)-*N′*-5-(4-chlorophenyl)-2-furoyl hydrazine (III-1).** Yield 86%, m.p. 174–175 °C. *R*_f_ = 0.22. IR (KBr) *ν*_max_: 3254.29 (hydrazine, N–H), 2874.93 (aromatic, C=C–H), 1635.34 (hydrazide, C=O), 1475.28, 1364.39, 1321.00 (aromatic, C=C), 1278.57, 1221.68 (aromatic, C–C and C–O), 1195.65, 1164.79, 1090.55, 1028.84 cm^−1. 1^H NMR (500 MHz, DMSO-*d*_6_) δ: 3.01 (t, 1H, *J* = 9.5 Hz, H-4), 3.05 (t, 1H, *J* = 8.5 Hz, H-2), 3.14–3.24 (m, 2H, H-3, H-5), 3.48 (dd, 1H, *J* = 11.5, 5.5 Hz, H-6_a_), 3.72 (dd, 1H, *J* = 11.5, 2.5 Hz, H-6_b_), 3.89 (d, 1H, *J* = 8.5 Hz, H-1), 7.17 (d, 1H, *J* = 3.5 Hz, FuH), 7.28 (d, 1H, *J* = 3.5 Hz, FuH), 7.54 (d, 1H, *J* = 9.0 Hz, PhH), 7.67 (d, 1H, *J* = 8.5 Hz, PhH), 7.90 (d, 1H, *J* = 8.5 Hz, PhH), 7.97 (d, 1H, *J* = 8.5 Hz, PhH). ESI-HRMS calcd. for C_17_H_19_ClN_2_O_7_: [M + H]^+^ 399.0959; Found: 399.0965.

**Aldehydo-d-Galactose-5-(4-chlorophenyl)-2-furoyl hydrazone (III-2).** Yield 81%, m.p. 204–205 °C. *R*_f_ = 0.23. IR (KBr) *ν*_max_: 3486.75, 3208.00 (hydrazone, N–H), 2848.79 (aromatic, C=C–H), 1664.27 (hydrazide, C=O), 1625.15 (hydrazone, C=N), 1554.34, 1475.28 (aromatic, C=C), 1375.96, 1302.68, 1222.65 (aromatic, C–C and C–O), 1182.15, 1083.80, 1053.91 cm^−1. 1^H NMR (500 MHz, DMSO-*d*_6_) δ : 3.35–3.40 (m, 2H, H-4, H-6_b_), 3.50–3.54 (m, 2H, H-3, H-6_a_), 3.70 (q, 1H, *J* = 6.5 Hz, H-5), 4.16 (d, 1H, *J* = 6.5 Hz, H-2), 7.16 (d, 1H, *J* = 3.5 Hz, FuH), 7.31 (d, 1H, *J* = 3.5 Hz, FuH), 7.52 (d, 2H, *J* = 9.0 Hz, PhH), 7.65 (d, 1H, *J* = 8.5 Hz, PhH), 7.85 (d, 1H, *J* = 6.0 Hz, CH=N), 7.93 (d, 1H, *J* = 8.0 Hz, PhH). ESI-HRMS Calcd. for C_17_H_19_ClN_2_O_7_: [M + H]^+^ 399.0959; Found: 399.0946.

**Aldehydo-d-Mannose-5-(4-chlorophenyl)-2-furoyl hydrazone (III-3).** Yield 78%, m.p. 192–193 °C. *R*_f_ = 0.19. IR (KBr) *ν*_max_: 3268.75 (hydrazone, N–H), 2938.67 (aromatic, C=C–H), 1677.77 (hydrazide, C=O), 1619.47 (hydrazone, C=N), 1551.45, 1473.35, 1409.71 (aromatic, C=C), 1298.82, 1275.68, 1222.65 (aromatic, C–C and C–O), 1173.47, 1075.12, 1028.84 cm^−1. 1^H NMR (500 MHz, DMSO-*d*_6_) δ: 3.36–3.40 (m, 1H, H-5), 3.43–3.48 (m, 1H, H-6a), 3.54 (t, 1H, *J* = 8.0 Hz, H-4), 3.59–3.62 (m, 1H, H-6b), 3.69 (t, 1H, *J* = 8.0 Hz, H-3), 4.05–4.09 (m, 1H, H-2), 7.17 (d, 1H, *J* = 3.5 Hz, FuH), 7.30 (d, 1H, *J* = 3.5 Hz, FuH), 7.52 (d, 1H, *J* = 8.5 Hz, PhH), 7.66 (d, 1H, *J* = 8.5 Hz, PhH), 7.75 (d, 1H, *J* = 6.5 Hz, CH=N), 7.86 (d, 1H, *J* = 8.0 Hz, PhH), 7.93 (d, 1H, *J* = 8.0 Hz, PhH). ESI-HRMS Calcd. for C_17_H_19_ClN_2_O_7_: [M + H]^+^ 399.0959; Found: 399.0968.

**Aldehydo-d-Fucose-5-(4-chlorophenyl)-2-furoyl hydrazone (III-4).** Yield 74%, m.p. 212–213 °C. *R*_f_ = 0.18. IR (KBr) *ν*_max_: 3485.69, 3225.36 (hydrazone, N–H), 2897.43 (aromatic, C=C–H), 1663.30 (hydrazide, C=O), 1620.21 (hydrazone, C=N), 1552.42, 1475.28 (aromatic, C=C), 1376.93, 1300.75, 1221.68 (aromatic, C–C and C–O), 1180.22, 1025.94 cm^–1. 1^H NMR (500 MHz, DMSO-*d*_6_) δ: 1.07 (d, 1H, *J* = 6.5 Hz, CH_3_), 3.26 (t, 1H, *J* = 8.0 Hz, H-4), 3.49 (t, 1H, *J* = 8.5 Hz, H-5), 3.86–3.89 (m, 1H, H-3), 4.34–4.36 (m, 1H, H-2), 7.17 (d, 1H, *J* = 3.5 Hz, FuH), 7.31 (d, 1H, *J* = 3.5 Hz, FuH), 7.52 (d, 2H, *J* = 8.5 Hz, PhH), 7.65 (d, 1H, *J* = 9.0 Hz, PhH), 7.84 (d, 1H, *J* = 6.5 Hz, CH=N), 7.93 (d, 1H, *J* = 8.0 Hz, PhH). ESI-HRMS Calcd. for C_17_H_19_ClN_2_O_6_: [M + H]^+^ 383.1010; Found: 383.1023.

**Aldehydo-d-Arabinose-5-(4-chlorophenyl)-2-furoyl hydrazone (III-5).** Yield 72%, m.p. 212–213 °C. *R*_f_ = 0.20. IR (KBr) *ν*_max_: 3442.31, 3076.18 (hydrazone, N–H), 2845.34 (aromatic, C=C–H), 1666.47 (hydrazide, C=O), 1627.63 (hydrazone, C=N), 1546.63, 1473.35 (aromatic, C=C), 1322.93, 1272.79 (aromatic, C–C and C–O), 1169.62, 1061.62, 1036.55 cm^–1. 1^H NMR (500 MHz, DMSO-*d*_6_) δ: 3.36–3.43 (m, 2H, H-3, H-5a), 3.49–3.54 (m, 1H, H-5b), 3.56–3.60 (m, 1H, H-4), 4.31–4.33 (m, 1H, H-2), 7.16 (d, 1H, *J* = 3.5 Hz, FuH), 7.30 (d, 1H, *J* = 3.5 Hz, FuH), 7.52 (d, 1H, *J* = 9.0 Hz, PhH), 7.65 (d, 1H, *J* = 8.5 Hz, PhH), 7.83 (d, 1H, *J* = 6.0 Hz, CH=N), 7.86 (d, 1H, *J* = 8.5 Hz, PhH), 7.93 (d, 1H, *J* = 8.5 Hz, PhH). ESI-HRMS Calcd. for C_16_H_17_ClN_2_O_6_: [M + H]^+^ 369.0853; Found: 369.0842.

***N*-(β-d-Glucopyranosyl)-*N′*-5-(2,4-difluorophenyl)-2-furoyl hydrazine (III-6).** Yield 75%, m.p. 184–185 °C. *R*_f_ = 0.23. IR (KBr) *ν*_max_: 3284.18 (hydrazine, N–H), 2931.26 (aromatic, C=C–H), 1655.59 (hydrazide, C=O), 1578.45, 1483.96 (aromatic, C=C), 1313.29, 1272.79 (aromatic, C–C and C–O), 1029.80 cm^–1. 1^H NMR (300 MHz, DMSO-*d*_6_) δ: 3.06–3.09 (m, 2H, H-2, H-4), 3.14–3.24 (m, 2H, H-3, H-5), 3.49 (dd, 1H, *J* = 11.7, 5.1 Hz, H-6_a_), 3.83 (dd, 1H, *J* = 12.0, 2.4 Hz, H-6_b_), 3.93 (d, 1H, *J* = 8.1 Hz, H-1), 6.96 (t, 1H, *J* = 3.6 Hz, FuH), 7.20–7.32 (m, 2H, FuH + PhH), 7.44–7.47 (m, 1H, PhH), 8.18–8.21 (m, 1H, PhH). ESI-HRMS Calcd. for C_17_H_18_F_2_N_2_O_7_: [M + H]^+^ 401.1160; Found: 401.1176.

***N*-(β-d-Glucopyranosyl)-*N′*-5-(4-fluorophenyl)-2-furoyl hydrazine (III-7).** Yield 84%, m.p. 200–201 °C. *R*_f_ = 0.22. IR (KBr) *ν*_max_: 3447.13 (hydrazine, N–H), 2933.20 (aromatic, C=C–H), 1635.34 (hydrazide, C=O), 1560.13, 1489.74 (aromatic, C=C), 1314.25, 1224.58 (aromatic, C–C and C–O), 1158.04, 1025.94 cm^–1. 1^H NMR (300 MHz, DMSO-*d*_6_) δ: 3.06–3.10 (m, 2H, H-2, H-4), 3.14–3.24 (m, 2H, H-3, H-5), 3.48 (dd, 1H, *J* = 12.3, 5.6 Hz, H-6_a_), 3.81 (dd, 1H, *J* = 12.2, 2.7 Hz, H-6_b_), 3.92 (d, 1H, *J* = 8.7 Hz, H-1), 7.14 (d, *J* = 3.6 Hz, 1H, FuH), 7.33 (d, *J* = 3.6 Hz, 1H, FuH), 7.37 (d, *J* = 9.0 Hz, 2H, PhH), 7.99–8.02 (m, 2H, PhH). ESI-HRMS Calcd. for C_17_H_19_FN_2_O_7_: [M + H]^+^ 383.1255; Found: 383.1267.

***N*-(β-d-Glucopyranosyl)-*N′*-5-(4-nitrophenyl)-2-furoyl hydrazine (III-8).** Yield 72%, m.p. 124–125 °C. *R*_f_ = 0.21. IR (KBr) *ν*_max_: 3344.93 (hydrazine, N–H), 2968.74 (aromatic, C=C–H), 1614.13 (hydrazide, C=O), 1486.85 (aromatic, C=C), 1332.57 (aromatic, C–C and C–O), 1078.01, 1021.12 cm^−1. 1^H NMR (300 MHz, DMSO-*d_6_*) δ: 3.06 (t, 1H, *J* = 9.6 Hz, H-4), 3.08 (t, 1H, *J* = 8.1 Hz, H-2), 3.18–3.24 (m, 2H, H-3, H-5), 3.49 (m, 1H, H-6_a_), 3.74 (m, 1H, H-6_b_), 3.91 (d, 1H, *J* = 8.5 Hz, H-1), 7.36 (d, 1H, *J* = 3.6 Hz, FuH), 7.58–7.63 (m, 3H, FuH + 2PhH), 8.30 (d, 2H, *J* = 9.0 Hz, PhH). ESI-HRMS Calcd. for C_17_H_19_N_3_O_9_: [M + H]^+^ 410.1200; Found: 410.1189.

***N*-(β-d-Glucopyranosyl)-*N′*-5-(2-nitrophenyl)-2-furoyl hydrazine (III-9).** Yield 69%, m.p. 133–134 °C. *R*_f_ = 0.23. IR (KBr) *ν*_max_: 3290.93 (hydrazine, N–H), 2897.86 (aromatic, C=C–H), 1646.91 (hydrazide, C=O), 1586.77, 1519.63, 1472.38 (aromatic, C=C), 1356.68, 1316.18, 1251.57 (aromatic, C–C and C–O), 1077.05 cm^−1. 1^H NMR (300 MHz, DMSO-*d*_6_) δ: 3.04–3.10 (m, 2H, H-2, H-4), 3.12–3.21 (m, 2H, H-3, H-5), 3.46 (m, 1H, H-6_a_), 3.80 (m, 1H, H-6_b_), 3.95 (d, 1H, *J* = 8.5 Hz, H-1), 6.90 (d, *J* = 3.5 Hz,1H, FuH), 7.34 (d, J = 3.5 Hz, 1H, FuH), 7.65–7.69 (m, 2H, PhH), 7.78–7.82 (m, 1H, PhH), 7.97–8.01 (m, 1H, PhH). ESI-HRMS Calcd. for C_17_H_19_N_3_O_9_: [M + H]^+^ 410.1200; Found: 410.1219.

***N*-(β-d-Glucopyranosyl)-*N′*-5-(2-chlorophenyl)-2-furoyl hydrazine (III-10).** Yield 81%, m.p. 160–161 °C. *R*_f_ = 0.21. IR (KBr) *ν*_max_: 3320.82 (hydrazine, N–H), 2925.48 (aromatic, C=C–H), 1654.62 (hydrazide, C=O), 1560.13, 1472.38 (aromatic, C=C), 1315.21(aromatic, C–C and C–O), 1073.19 cm^–1. 1^H NMR (300 MHz, DMSO-*d*_6_) δ: 3.01 (t, 1H, *J* = 9.6 Hz, H-4), 3.06 (t, 1H, *J* = 8.7 Hz, H-2), 3.12–3.24 (m, 2H, H-3, H-5), 3.46 (dd, 1H, *J* = 12.6, 5.1 Hz, H-6_a_), 3.82 (dd, 1H, *J* = 12.6, 2.7 Hz, H-6_b_), 3.92 (d, 1H, *J* = 8.4 Hz, H-1), 7.31 (d, J = 3.6 Hz, 1H, FuH), 7.39 (d, *J* = 3.6 Hz, 1H, FuH), 7.43–7.54 (m, 2H, PhH), 7.60–7.63 (m, 1H, PhH), 8.18–8.22 (m, 1H, PhH). ESI-HRMS Calcd. for C_17_H_19_ClN_2_O_7_: [M + H]^+^ 399.0959; Found: 399.0968.

***N*-(β-d-Glucopyranosyl)-*N′*-5-(4-methylphenyl)-2-furoyl hydrazine (III-11).** Yield 79%, m.p. 180–181 °C. *R*_f_ = 0.23. IR (KBr) *ν*_max_: 3456.78 (hydrazine, N–H), 2906.20 (aromatic, C=C–H), 1638.23 (hydrazide, C=O), 1577.49, 1491.67, 1454.06 (aromatic, C=C), 1313.29, 1271.82 (aromatic, C–C and C–O), 1078.01, 1018.23 cm^–1. 1^H NMR (300 MHz, DMSO-*d*_6_) δ: 2.34 (s,3H,CH_3_), 3.02 (t, 1H, *J* = 9.6 Hz, H-4), 3.06 (t, 1H, *J* = 8.1 Hz, H-2), 3.16–3.23 (m, 2H, H-3, H-5), 3.49 (dd, 1H, *J* = 12.3, 5.1 Hz, H-6_a_), 3.69 (dd, 1H, *J* = 12.2, 2.7 Hz, H-6_b_), 3.95 (d, 1H, *J* = 8.8 Hz, H-1), 7.02 (d, 1H, *J* = 3.6 Hz, FuH), 7.27–7.33 (m, 3H, FuH +2PhH), 7.78–7.83 (m, 2H, PhH). ESI-HRMS Calcd. for C_18_H_22_N_2_O_7_: [M + H]^+^ 379.1505; Found: 379.1516.

***N*-(β-d-Glucopyranosyl)-*N′*-5-(3-fluorophenyl)-2-furoyl hydrazine (III-12).** Yield 78%, m.p. 184–185 °C. *R*_f_ = 0.22. IR (KBr) *ν*_max_: 3380.60 (hydrazine, N–H), 2879.62 (aromatic, C=C–H), 1660.41 (hydrazide, C=O), 1587.13, 1473.35, 1412.60 (aromatic, C=C), 1316.18 (aromatic, C–C and C–O), 1194.69, 1074.16, 1028.84 cm^−1. 1^H NMR (300 MHz, DMSO-*d*_6_) δ: 3.02–3.10 (m, 2H, H-2, H-4), 3.12–3.21 (m, 2H, H-3, H-5), 3.43 (dd, 1H, *J* = 12.3, 5.1 Hz, H-6_a_), 3.76 (dd, 1H, *J* = 12.6, 2.7 Hz, H-6_b_), 3.92 (d, 1H, *J* = 8.4 Hz, H-1), 7.26–7.31 (m, 2H, FuH + PhH), 7.45 (d, *J* = 3.6 Hz, 1H, FuH), 7.55–7.58 (m, 1H, PhH), 7.74–7.77 (m, 1H, PhH), 7.98–8.01 (m, 1H, PhH). ESI-HRMS Calcd. for C_17_H_19_FN_2_O_7_: [M + H]^+^ 383.1255; Found: 383.1264.

### 3.3. Bioassay

#### 3.3.1. Fungicidal Activity

The fungicidal activity and antifungal spectra of title compounds were evaluated using the mycelium growth rate test at 50 μg·mL^−1^. The fungi were provided and maintained by Department of Applied Chemistry, College of Science, China Agricultural University, Beijing, China. The commercial fungicides mancozeb, carbendazim, thiram, and hymexazol were used as positive controls. EXCEL2007 was applied to analyze bioassay data. Analysis of difference significance was performed using SPSS v.18.0 (SPSS Inc., Chicago, IL, USA).

The fungicidal activity of title compounds was assessed using the radial growth test on potato dextrose agar (PDA). The compounds were dissolved in acetone and mixed with sterile molten PDA to obtain different concentrations of 50, 25, 12.5, 6.25, 3.13, 1.56 and 0.78 μg·mL^−1^. Three replicates were used per treatment. After an incubation period of 96 h at 23 °C under a regular 12:12 h light:dark regimen, mycelial growth diameters were measured and the inhibition percentages relative to the control with 1% acetone were calculated. The inhibition rate was calculated according to the following formula:

(1) $$I_{1}=({\bar{D}}_{1}-{\bar{D}}_{0})/{\bar{D}}_{1}\times 100\%$$

where *I*_1_ is the inhibition rate, *D̄*_1_ is the average area of mycelia in the blank test, and *D̄*_0_ is the average area of mycelia in the presence of compounds. The EC_50_ and EC_80_ values were calculated using log-probit analysis, and the results were given in Table 1.

#### 3.3.2. Antitumor Activity

All the title compounds were dissolved in DMSO and screened for preliminary anticancer activity against four different cell lines: a human promyelocytic leukemic cell line (HL-60), a human hepatocellular carcinoma cell line (Bel-7402), a human gastric carcinoma cell line (BGC-823), and a human nasopharyngeal carcinoma cell line (KB) at concentration gradient of 0.1, 1.0, 10, 50 and 100 μM. The commercial drug doxorubicin was used as positive control in the bioassay. Three replicates were performed.

The four types of cell line were grown and maintained in RPMI-1640 medium supplemented with 10% fetal bovine serum (FBS), penicillin (100 U·mL^−1^), and streptomycin (100 μg·mL^−1^) at 37 °C in humidified incubators in an atmosphere of 5% CO_2_.

All the experiments were performed on exponentially growing cancer cells. Numbers of viable cancer cells were determined by MTT ([3-(4,5-dimethylthiazol-2-yl)-2,5-diphenyl tetrazoliumbromide]) [30] and SRB (sulforhodamine B) [31] assays. The cancer cells (1 ~ 2.5 × 10^4^ cells·mL^−1^) were inoculated in 96-well culture plates (180 μL/well). After 24 h, 20 μL of culture medium containing compounds of various concentrations were added to the wells and, the cells were incubated for 48 h. 20 μL of RPMI-1640 medium was added to the control cells. HL-60 cells were assayed by MTT, and the Bel-7402, BGC-823 and KB cells were assayed by SRB. The absorbance of each well was measured using a microculture plate reader at 570 nm (MTT) and 540 nm (SRB). The inhibition rate was calculated according to the following formula:

(2) $$\text{Inhibition rate}=(OD_{\text{control}}-{\text{OD}}_{\text{treated}})/{\text{OD}}_{\text{control}}\times 100\%$$

## 4. Conclusions

In summary, a novel series of *N*-glycosyl-*N′*-(5-substituted phenyl-2-furoyl) hydrazide derivatives were synthesized in good yields. The anomerization and ring-chain isomers were confirmed by ^1^H NMR spectroscopy. Their antifungal and antitumor tests indicated that some title compounds showed superiority over the commercial positive controls during the present studies. Comparing title compounds **III** with lead compounds **B** (diacylhydrazides), **C** (semicarbazides), and **D** (acylhydrazones) (Scheme 1), it can be seen that the modification with sugars in hydrazide molecules is important for improving the antifungal and antitumor activity. These compounds could be lead compounds for further development.

## Figures and Tables

**Table 1.** ^1^H NMR spectra data of the condensation products of 5-(4′-chlorophenyl)-2-furoyl hydrazide with different monosaccharides.

Compounds	Monosaccharide	Chemical Shift (500 MHz, DMSO-*d*_6_)			Tautomer

		H-1	NH-1	NH-2	
**III-1**	Glc	3.89 (t, 8.5 Hz)	5.87 (t, 4.0 Hz)	10.18 (s)	β*-*pyronose
**III-2**	Gal	7.85 (d, 6.0 Hz)	–	11.52 (s)	acyclic
**III-3**	Man	7.75 (d, 6.5 Hz)	–	11.54 (s)	acyclic
**III-4**	Fuc	7.84 (d, 6.5 Hz)	–	11.52 (s)	acyclic
**III-5**	Ara	7.83 (d, 6.0 Hz)	–	11.53 (s)	acyclic

**Table 2.** The preliminary bioassay against 19 fungi at 50 μg·mL^−1^.

Fungi	Inhibitory Rate (%) ± SEM											

	III-1	III-2	III-3	III-4	III-5	III-6	III-7	III-8	III-10	III-11	III-12	III-13
*Fusarium graminearum*	–	23.44 ± 4.24	–	–	–	–	43.75 ± 4.96	–	–	–	–	–
*Pyricularia oryzae*	–	30.24 ± 4.59	–	–	–	–	26.39 ± 4.41	–	64.41 ± 4.79	35.82 ± 4.79	–	–
*Phytophthora infestans*	–	90.16 ± 2.98	73.24 ± 4.43	26.48 ± 4.41	77.48 ± 4.18	87.80 ± 3.27	–	41.67 ± 4.93	–	63.90 ± 4.80	38.20 ± 4.86	–
*Alternaria alternata*	54.24 ± 4.98	58.13 ± 4.93	65.40 ± 4.76	–	–	32.18 ± 4.67	–	41.52 ± 4.93	–	22.15 ± 4.15	–	58.13 ± 4.93
*Valsa mali*	41.64 ± 4.93	45.82 ± 4.98	46.94 ± 4.99	–	46.20 ± 4.99	79.13 ± 4.06	–	40.86 ± 4.92	45.07 ± 4.98	–	36.08 ± 4.80	53.73 ± 4.99
*Colletotrichum gloeosporioides*	–	–	–	–	24.92 ± 4.33	34.45 ± 4.75	40.29 ± 4.90	–	32.12 ± 4.67	32.12 ± 4.67	–	36.73 ± 4.82
*Monilinia ariae*	–	–	–	39.31 ± 4.88	33.75 ± 4.73	–	–	–	–	–	21.90 ± 4.14	35.63 ± 4.79
*Gloeosporium musarum*	–	21.75 ± 4.13	–	20.84 ± 4.06	34.76 ± 4.76	21.75 ± 4.13	30.13 ± 4.59	–	–	–	–	30.56 ± 4.61
*Fusarium oxysporum* f. sp. Niveum	–	29.94 ± 4.58	36.60 ± 4.82	27.94 ± 4.49	31.35 ± 4.64	–	–	–	25.03 ± 4.33	–	28.80 ± 4.53	24.73 ± 4.31
*Botrytis cinerea* Pers.	–	–	28.09 ± 4.49	–	–	–	–	–	–	–	–	21.15 ± 4.08
*Colletotrichum orbiculare*	–	–	–	42.45 ± 4.94	25.68 ± 4.37	–	38.73 ± 4.87	55.25 ± 4.97	–	21.46 ± 4.11	–	–
*Phytophthora melonis*	58.03 ± 4.94	–	–	–	32.12 ± 4.67	31.03 ± 4.63	29.27 ± 4.55	–	–	–	32.55 ± 4.69	–
*Sclerotinia sclerotiorum*	–	70.84 ± 4.54	–	–	–	32.76 ± 4.69	–	–	–	–	–	–
*Phomopsis aspamgi*	–	64.71 ± 4.78	25.44 ± 4.36	69.36 ± 4.61	55.39 ± 4.97	–	43.89 ± 4.96	91.07 ± 2.85	31.07 ± 4.63	47.16 ± 4.99	67.29 ± 4.69	68.95 ± 4.63
*Alternaria dauci*	20.09 ± 4.01	28.01 ± 4.49	–	37.92 ± 4.85	28.01 ± 4.49	22.77 ± 4.19	–	22.77 ± 4.19	–	22.77 ± 4.19	–	–
*Fusarium graminearum*	55.56 ± 4.97	–	24.54 ± 4.30	–	–	–	–	26.28 ± 4.40	–	–	–	–
*Cladosporium fulvum*	–	47.24 ± 4.99	42.81 ± 4.95	35.84 ± 4.80	54.22 ± 4.98	70.05 ± 4.58	–	50.09 ± 5.00	45.78 ± 4.98	42.81 ± 4.95	–	66.69 ± 4.71
*Colletotrichum phomoides*	26.53 ± 4.41	–	48.98 ± 5.00	48.98 ± 5.00	–	–	26.53 ± 4.41	38.27 ± 4.86	–	–	–	–
*Alternaria tenuis* Nees	21.84 ± 4.13	–	31.76 ± 4.66	24.39 ± 4.29	43.21 ± 4.95	31.76 ± 4.66	24.39 ± 4.29	–	–	41.00 ± 4.92	–	–

– means no activity.

**Table 3.** Fungicidal activity of title compounds.

Fungi	Compounds	*R*	Regression Equation	EC_50_ (95% Confidence Interval)/μg·mL^−1^	EC_80_ (95% Confidence Interval)/μg·mL^−1^
*P. infestans*	**III-2**	0.987	*y* = 2.283*x* – 10.678	5.476 (4.857~6.132)	456.337 (405.314~511.740)
	**III-5**	0.988	*y* = 2.294*x* – 10.715	5.695 (5.107~6.335)	485.901 (435.433~540.053)
	**III-6**	0.999	*y* = 1.950*x* – 9.10	4.493 (4.426~4.507)	195.839 (193.750~197.278)
	mancozeb	0.969	*y* = 1.923*x* – 8.973	4.385 (3.329~5.776)	182.460 (138.293~239.924)

*A. tenuis* Nees	**III-11**	0.979	*y* = 2.191*x* – 10.165	6.181 (5.111~7.437)	431.342 (356.891~519.335)
	thiram	0.976	*y* = 2.183*x* – 9.969	8.831 (8.144~9.574)	608.260 (559.935~658.234)

*C. gloeosporioides*	**III-7**	0.991	*y* = 1.932*x* – 8.964	4.962 (4.580~5.384)	210.254 (193.593~227.579)
	carbendazm	0.993	*y* = 2.237*x* – 10.521	4.613 (4.331~4.912)	352.820 (330.646~375.017)

*P. aspamgi*	**III-7**	0.977	*y* = 1.732*x* – 8.222	2.737 (2.232~3.366)	78.767 (64.0567~96.5630)
	hymexazol	0.985	*y* = 2.018*x* – 9.527	3.656 (2.232~3.366)	78.770 (64.057~96.563)

*P. melonis*	**III-1**	0.985	*y* = 2.427*x* – 11.254	7.586 (6.647~8.696)	840.493 (733.256~959.342)
	**III-9**	0.996	*y* = 2.354*x* – 11.053	5.179 (5.027~5.403)	500.113 (481.424~517.398)
	mancozeb	0.991	*y* = 1.892*x* – 8.727	5.408 (4.987~5.863)	211.870 (195.086~229.334)

**Table 4.** Antitumor activity of the title compounds.

Compounds	IC_50_ (μM)			

	HL-60	BGC-823	Bel-7402	KB
**III-1**	267.9	184.6	2.6 × 10^5^	586.2
**III-2**	167.0	90.9	133.4	166.8
**III-3**	6.8	151.9	144.5	9.0
**III-4**	1.2	34.0	169.9	31.9
**III-5**	19.4	47.2	106.8	118.9
**III-6**	31.7	43.5	110.4	40.7
**III-7**	78.9	193.3	356.7	489.2
**III-8**	61.9	96.1	258.0	116.6
**III-9**	38.7	550.2	178.3	337.8
**III-10**	1523.4	92.8	174.8	1.2 × 10^5^
**III-11**	5797.3	119.3	2.5 × 10^4^	100.7
**III-12**	315.1	76.2	3.5 × 10^6^	1588.0
**doxorubicin**	28.4	8.5	6.7	11.9
